# Assuming the right approach in cerebrofacial arteriovenous metameric syndrome: a case report

**DOI:** 10.3325/cmj.2026.66.456

**Published:** 2025-12

**Authors:** Maša Malenica, Vladimir Kalousek, Ana Smolić, Monika Kukuruzović, Tena Trbojević, Tomislav Sajko

**Affiliations:** 1 Department of Pediatrics, Sestre Milosrdnice University Hospital Center, Zagreb, Croatia; 2 European Reference Network EpiCARE; 3 Department of Radiology, Sestre Milosrdnice University Hospital Center, Zagreb Croatia; 4 Practice of General Medicine Dr Mladena Strukan, Zagreb, Croatia; 5 Department of Neurosurgery, Sestre Milosrdnice University Hospital Center, Zagreb, Croatia

Cerebrofacial arteriovenous metameric syndrome (CAMS) is a rare nonhereditary disease, amounting to only 0.5% of all cerebral vascular malformations. It is characterized by vascular malformations in a metameric distribution involving the cerebrofacial region. We present a case of a 14-year-old girl in whom the first vascular lesion appeared on the tip of her nose at the age of 18 months. This lesion reoccurred on several occasions during her childhood, with exacerbations coinciding with common rhinitis and several nose bleeds. She was unsuccessfully treated with laser therapy and topical selective beta-blockers prior to further investigations. Combined cerebrofacial arteriovenous malformation (CAMS I+II) was diagnosed by magnetic resonance imaging of the paranasal sinuses, orbits, and brain with cerebral angiography and confirmed by digital subtraction angiography. Clarifying the correct diagnosis enabled us to abort a potential surgical approach and to assume an expectant approach unless the patient develops neurological symptoms. Timely, complete, and adequate imaging investigations are necessary in cases of facial vascular malformations. This report for the first time describes the specific angioarchitecture involving two types of CAMS.

Cerebrofacial arteriovenous metameric syndrome (CAMS) is characterized by metameric distribution of vascular malformations involving the craniofacial region, with three subtypes based on the location of lesions (1). Due to the rarity of the syndrome and the small size of skin lesions, initial management could be misled. Herein, we report, for the first time, a case of a facial vascular lesion located on the tip of the nose. Adequate neuroimaging showed the lesion to be a rare combination of CAMS I+II, guiding our therapeutic plan to an expectant course.

## Case report

**Figure 1:**
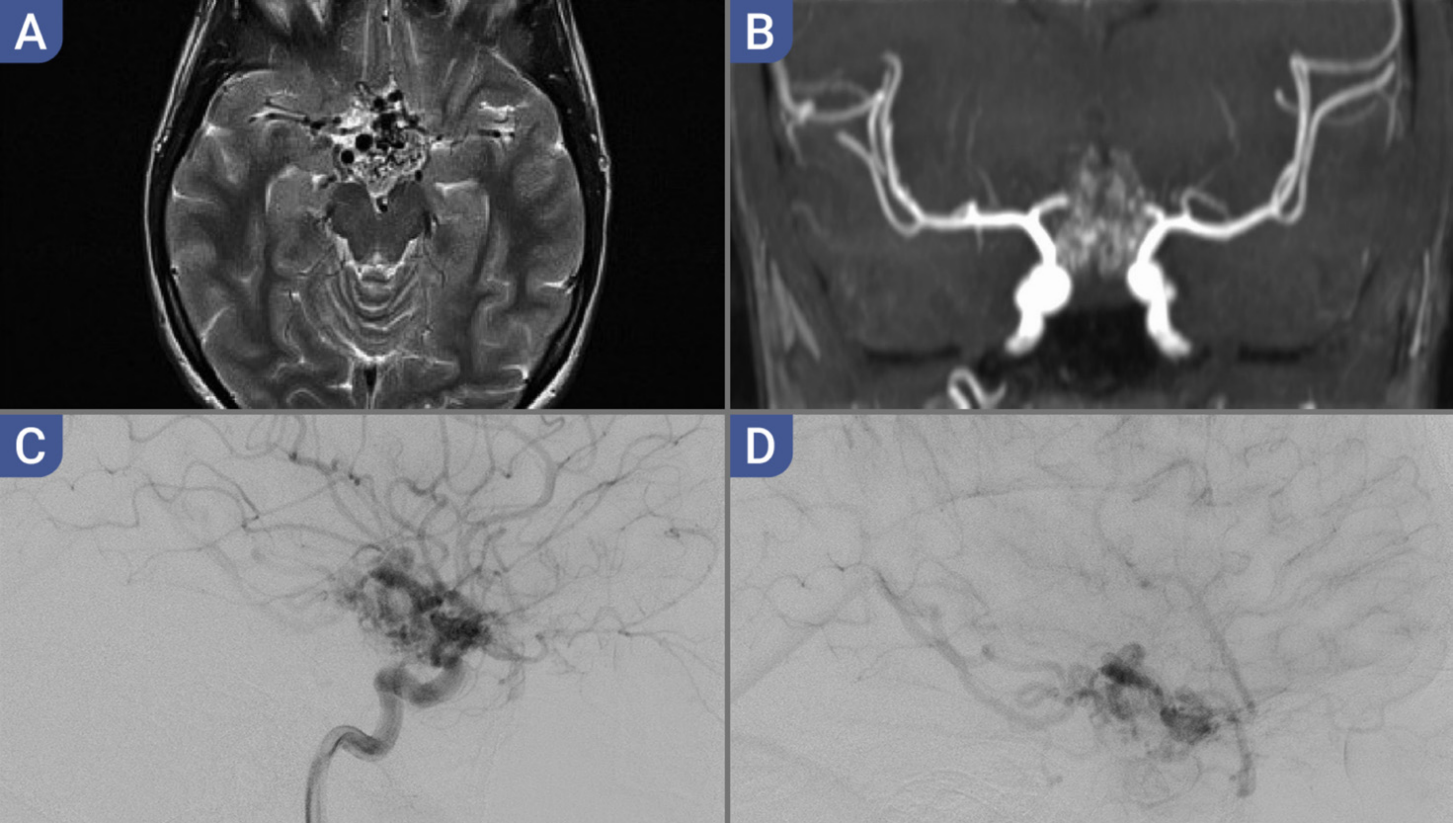
(**A**) Magnetic resonance imaging (MRI) of the brain with angiography 1.5T T2 transversal. (**B**) MR angiography of the brain 1.5T 3D time-of-flight maximum intensity projection reconstruction. (**C**) Digital subtraction angiography latero-lateral projection of the right internal carotid artery (ICA) arterial phase. (**D**) Digital subtraction angiography right ICA injection latero-lateral venous phase.

**Figure 2:**
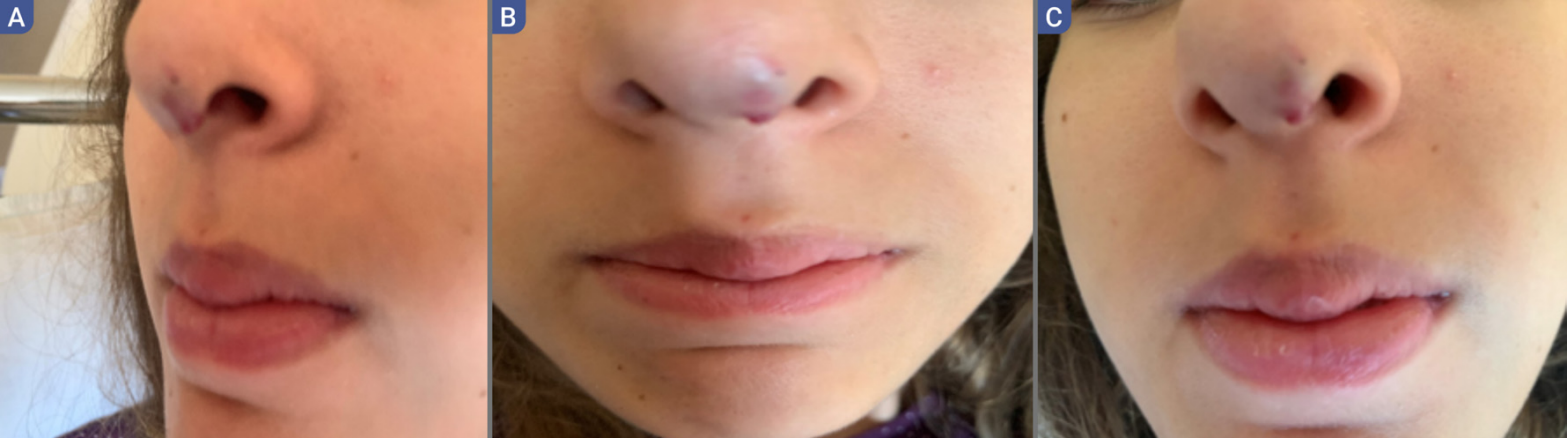
(**A**-**C**) Clinical presentation of our patient with a visible angiomatous lesion at the tip of the nose.

We report on a case of a 14-year-old girl with no family history of vascular malformations. At the age of 18 months, a small angiomatous lesion was noted at the tip of her nose, which disappeared after a few months. At the age of 10 years, the lesion re-appeared, increased in size with a small hemorrhage related to an upper respiratory tract infection. Initially, dermatologists treated the lesion with laser therapy (I2PL), which resulted in a moderate decrease in size. Further therapy with coagulation using an Nd-YAG laser yielded no success. Later, upon a dermatologist’s suggestion, the patient was somewhat successfully treated with a topical 0.5% solution of a nonselective beta-blocker. Differential diagnosis primarily included pyogenic granuloma due to presumable reactive proliferation of capillary blood vessels, or occasional bleeding, which is why the patient was referred to an otorhinolaryngology (ENT) clinic. Magnetic resonance imaging (MRI) of the paranasal sinuses, orbits, and brain with angiography using the 3D time of flight (TOF) technique revealed arteriovenous malformation (AVM) in the suprasellar cisternae very close to the optic chiasm. Feeding arteries were not identifiable with certainty, but venous drainage was dominantly into slightly dilated Rosenthal veins. In the nasal area, there was postcontrast imbibition with a prominent small artery – possibly a hemangioma (Figure 1A, 1B). Selective cerebral digital subtraction angiography (DSA) demonstrated AVM with multiple feeders from the periophtalmic segments of both carotid arteries and from both A1 complexes (Figure 1C). The nidus of the AVM was drained via Rosenthal veins into the sinus rectus (Figure 1D). Upon examination, the angiomatous lesion was soft, non-pulsatile, reddish-purple, and 10 × 10 mm in size with a growth within it sized 5 × 5 mm, elevated from the surrounding skin (Figure 2A, 2B, 2C). Dermatoscopically, it was a vascular lesion corresponding to imaging findings. Based on the physical finding and the distribution of the craniofacial venous malformations, as well as a review of published literature, we diagnosed two combined types of CAMS (I+II) (Table 1). After making the diagnosis, the initially planned surgical procedure was called off. In view of her mild clinical symptoms, the patient is currently being managed conservatively unless she develops neurological symptoms.

**Table 1:** The timeline of events

Year	Clinical data
2009	Hemangioma became visible on the tip of patient’s nose
2011	Hemangioma disappeared
2014	Hemangioma re-appeared and spontaneously bled with respiratory infections
2017	Hemangioma shrunk after a significant bleed
2018	MR of the paranasal sinuses: arteriovenous malformation in the area of suprasellar cisternae
2019	MR DSA: arteriovenous malformation resembling CAMS type II
2020	Additional MR angiography describes CAMS type I and II
2023	Neuroradiological follow-up was stationary
2024	Transition to adult neurologist

*Abbreviations: MR – magnetic resonance; DSA – digital subtraction angiography; CAMS – cerebrofacial arteriovenous metameric syndrome.

## Discussion

CAMS is a rare, typically unilateral condition characterized by AVMs affecting the cerebral, retinal, and facial areas (2). It is categorized into three types based on the location of lesions: CAMS I – medial prosencephalic group involving the nose and hypothalamus; CAMS II – lateral prosencephalic group involving the occipital lobe, optic chiasma, optic tract, thalamus, retina, and maxilla; and CAMS III – rhombencephalic group involving the cerebellum, pons, and mandible (3). The pathogenesis of this complex vascular malformation composed of intracranial and extracranial lesions, also known as the Wyburn-Mason or Bonnet-Dechaume-Blanc syndrome (4), has been difficult to explain. The metameric vascular lesions are thought to be produced by a somatic mutation or an early embryonic vascular insult in the neural crest prior to migration, which may lead to migration anomalies with segmental distribution linking the brain and the face as seen in CAMS (2). Clinical manifestation varies depending on organ involvement. While mostly having a silent course, the disease can present with progressive visual loss, neurological deficit, potential hemorrhaging, and seldom seizures (5,6). Associated facial vascular malformations are considered uncommon and hence can be difficult to recognize. They could also be clinically silent for long periods, as in our patient, sometimes presenting as a small red spot or angioma since infancy or early childhood without a clear cause of exacerbation (7). A proper diagnosis must rely on neuroimaging, initially MRI and angiography, which provide high specificity and negative predictive value for cerebrofacial AVMs. Upon discovery of an AVM in the cerebrofacial region, other clinically silent AVMs should be searched for along the same metameric level (5). The importance of our case lies in the extremely rare, intermittent presentation of a vascular lesion on the tip of the nose, previously published only in a series by Bhattacharija (3) and in a report by Jiarakongmun et al (6). In addition, this is the first published case of the two types of CAMS (I+II) presenting together. Due to its esthetic effect and the absence of neurologic symptoms, the AVM in our case was initially exclusively treated by a dermatologist. The patient was then referred to ENT specialists for histology specimen sampling despite previous hemorrhage from the lesion. Treatment algorithms in CAMS vary considerably and may reflect the degree and location of organ involvement, and in cases of only esthetic concern, this may mislead the physician. The current recommendation is management by a multidisciplinary team (8). While most of intracranial AVMs are considered incurable and are treated through conservative management (5), in specific cases embolization therapy has produced satisfactory results. Jiarakongmun et al reported successful endovascular embolization of not only intracranial, but also facial AVMs (6). A treatment strategy for facial AVMs could be based on the preservation of function along with cosmetic and reconstructive considerations. In such cases, a combined approach involving endovascular therapy and reconstructive plastic surgery is advised to achieve optimal outcomes. However, in view of the risk management of potential hemorrhage and poor outcome, the typical course in the absence of neurological symptoms in CAMS I+II patients, as shown in our case, is conservative management with a careful follow-up.

Facial vascular lesions are an extremely rare presentation of CAMS and can be difficult to recognize. Nevertheless, knowledge of the syndrome is crucial and should gear the physician toward neuroimaging, thereby ensuring the right diagnosis is made by MRI of the brain and angiography. In our case, the facial vascular lesion extends along the chiasm, which makes it inoperable and requires an expectant course, unless a neurologic deficit prompts more aggressive action. The importance of this case report lies in recognizing the syndrome and assuming the right treatment approach.
